# Characterization and Immobilization of a Novel SGNH Family Esterase (*La*SGNH1) from *Lactobacillus acidophilus* NCFM

**DOI:** 10.3390/ijms21010091

**Published:** 2019-12-21

**Authors:** Ly Thi Huong Luu Le, Wanki Yoo, Sangeun Jeon, Kyeong Kyu Kim, T. Doohun Kim

**Affiliations:** 1 Department of Chemistry, College of Natural Science, Sookmyung Women’s University, Seoul 04310, Korea; ly.12000532@gmail.com (L.T.H.L.L.); vlqkshqk61@outlook.kr (W.Y.); sangeun94@sookmyung.ac.kr (S.J.); 2 Department of Precision Medicine, Samsung Biomedical Research Institute, Sungkyunkwan University School of Medicine, Suwon 440-746, Korea; kyeongkyu@skku.edu

**Keywords:** *La*SGNH1, *Lactobacillus acidophilus*, SGNH family esterases, immobilization, crosslinked enzyme aggregates

## Abstract

The SGNH family esterases are highly effective biocatalysts due to their strong catalytic efficiencies, great stabilities, relatively small sizes, and ease of immobilization. Here, a novel SGNH family esterase (*La*SGNH1) from *Lactobacillus acidophilus* NCFM, which has homologues in many *Lactobacillus* species, was identified, characterized, and immobilized. *La*SGNH1 is highly active towards acetate- or butyrate-containing compounds, such as *p*-nitrophenyl acetate or 1-naphthyl acetate. Enzymatic properties of *La*SGNH1, including thermal stability, optimum pH, chemical stability, and urea stability, were investigated. Interestingly, *La*SGNH1 displayed a wide range of substrate specificity that included glyceryl tributyrate, *tert*-butyl acetate, and glucose pentaacetate. Furthermore, immobilization of *La*SGNH1 by crosslinked enzyme aggregates (CLEAs) showed enhanced thermal stability and efficient recycling property. In summary, this work paves the way for molecular understandings and industrial applications of a novel SGNH family esterase (*La*SGNH1) from *Lactobacillus acidophilus*.

## 1. Introduction

Lipolytic enzymes such as (phospho)lipases or esterases, which are present throughout three domains of life (Eukarya, Bacteria, and Archaea), are generally involved in the hydrolysis of lipids or their derivatives [1,2,3]. They share similar structural and catalytic features, including a highly conserved catalytic triad (Ser-Asp/Glu-His), an α/β hydrolase fold, broad substrate specificity, and an absence of cofactors [4,5]. Among them, enzymes of microbial origin have been extensively used in a wide variety of applications, such as pharmaceutical, fine chemical, and food industries. They displayed excellent stability, high efficiency, and strong stereoselectivity [6,7]. Recently, SGNH family esterases have attracted interest because they are highly useful for the preparation of aromas, flavors, drug intermediates, and pharmaceutical products [8,9,10]. They are characterized by four conserved sequence blocks of I–III and V in their primary sequences [8,9]. In these enzymes, the catalytic serine is located in the highly conserved motif of Gly-Asp-Ser (GDS) in the *N*-terminal region. In addition, Gly and Asn in motif II and III are responsible for the formation of a tetrahedral intermediate and an oxyanion hole. The Asp-x-x-His tetrapeptide in motif V constitutes the catalytic machinery of these enzymes. To date, a number of SGNH family esterases have been identified and characterized from several microorganisms [11,12,13,14,15,16,17,18,19], but there are very few reports in lactic acid bacteria.

*Lactobacillus acidophilus* is one of the most widely used industrial microorganisms in the bioprocessing of dairy products, fermented food, and nutritional and dietary supplements [20,21,22]. In addition, *L. acidophilus* can produce a number of antimicrobial peptides, organic metabolites and acids, and vitamins through diverse metabolic processes. The production of these molecules is largely responsible for the stimulation of inherent immune systems and the reduction of pathological inflammations [23,24]. Therefore, this bacterium could be used as a rich and unique source for the identification of a large variety of enzymes with novel functions or characteristics.

Although several esterases have been described in *L. acidophilus*, no studies have been reported regarding SGNH family esterases [25,26]. Here, characterization and immobilization of a novel SGNH family esterase (NCBI Reference Sequence: WP_125978798, *La*SGNH1) from *L. acidophilus* NCFM were investigated. To our knowledge, this study is the first report on the SGNH family esterase from *L. acidophilus*.

## 2. Results and Discussion

### 2.1. Bioinformatic Analysis of LaSGNH1

**Figure 1:**
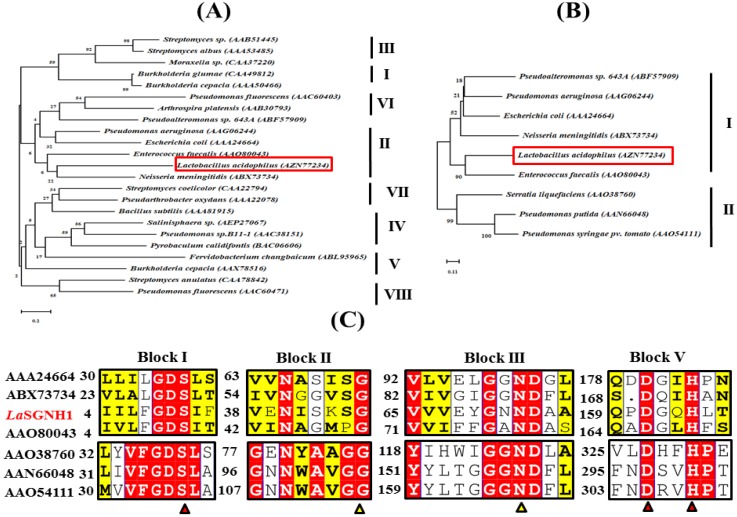
Phylogenetic tree and sequence analysis of *La*SGNH1. (**A**) Bacterial lipases/esterases family I–VIII, and (**B**) clade I and II of family II are shown. A red box in each panel indicates the location of *La*SGNH1. The phylogenetic trees were constructed with MEGA v. 7.0 using the neighbor-joining method, and all sequences were retrieved from the NCBI database. (**C**) Sequence alignments of four conserved blocks (Block I, II, III, and V) are shown, and highly conserved residues are highlighted in red. Sequences are aligned with Clustal Omega and ESPript. Highly conserved catalytic triad, glycine, and asparagine are shown as red or yellow triangles. Four sequences of the clade I subfamily are shown in the upper region, while three sequences of the clade II subfamily are shown in the bottom region. Highly important amino acids for catalysis are shown as red and yellow triangles.

In the chromosome of *L. acidophilus*, a gene encoding a novel SGNH family esterase (*La*SGNH1, locus tag: AZN77234, 561 bp) was identified using in silico bioinformatic analysis. Sequence analysis revealed that *La*SGNH1 had a molecular mass of ~21 kDa with a single polypeptide chain of 188 amino acids, with a pI of 5.93. For phylogenetic tree analysis, eight bacterial lipases/esterases families (I–VIII) were investigated (Figure 1A). *La*SGNH1 was shown to be a member of family II lipases/esterases, which is further subdivided into clade I and clade II subfamilies [25]. More specifically, as shown in Figure 1B, *La*SGNH1 was clustered in the clade I subfamily with a lipase/acylhydrolase from *Enterococcus faecalis* (AAO80043, 30.4% sequence identity).

As shown in Figure 1C, four blocks (I, II, III, and V) are highly conserved of *La*SGNH1 in clade I and II of family II lipases/esterases. The catalytic Ser^10^ is shown to be located in a GDS motif in block I, while a DXXH motif was localized in block V. Conserved residues in block II and III were shown to be involved in the formation of an oxyanion hole [8,25].

**Figure 2:**
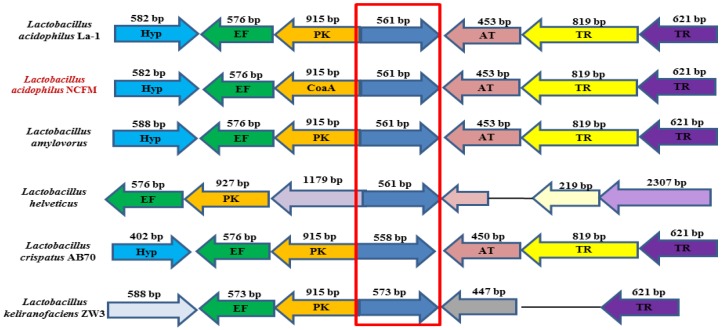
Gene cluster analysis of *La*SGNH1. Similar gene clusters were found in Lactobacillus species including in *Lactobacillus acidophilus* La-1, *L. acidophilus* NCFM, *Lactobacillus amylovorus*, *Lactobacillus helveticus*, *Lactobacillus crispatus* AB70, and *Lactobacillus kefiranofaciens* ZW3. EF: elongation factor, PK: type I pantothenate kinase, AT: acetyltransferase, TR: amino acid ABC transporter. Homologous proteins of *La*SGNH1 are shown in the red box.

Genomic cluster analysis revealed that *La*SGNH1 has homologues in other *Lactobacillus* species, including *Lactobacillus amylovorus*, which implies the invariant and important roles of these enzymes in *Lactobacillus* species (Figure 2). To date, there are no reports on these proteins, and their functional properties are largely unknown. The molar percentage (30.7%) of four hydrophobic amino acid residues (Alanine (Ala), Valine (Val), Isoleucine (Ile), and Leucine (Leu)) in *La*SGNH1, which was shown to be important for protein stability [26], is comparable to that of an SGNH hydrolase (LI22) from *Listeria innocua* [18] or a SGNH hydrolase (Est24) from *Sinorhizobium meliloti* [19].

### 2.2. Characterizations of LaSGNH1

**Figure 3:**
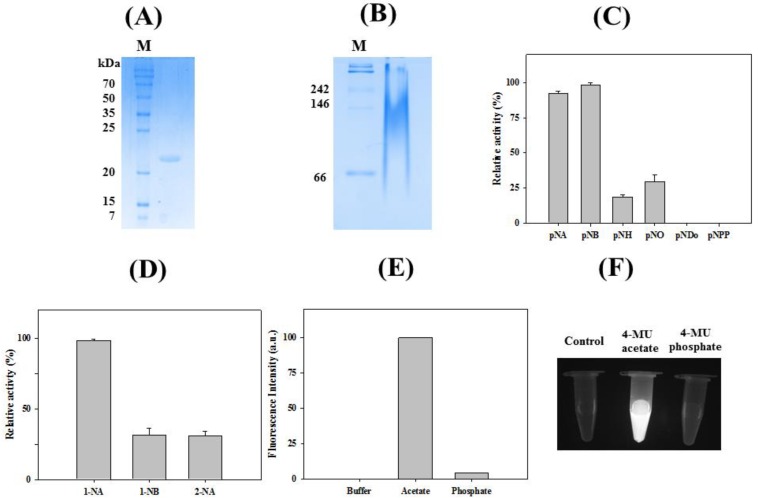
Characterization of *La*SGNH1. (**A**) Sodium dodecyl (lauryl) sulfate-polyacrylamide gel electrophoresis (SDS-PAGE) analysis of purified *La*SGNH1. (**B**) Native-polyacrylamide gel electrophoresis (PAGE) analysis. (**C**) Substrate specificity of *La*SGNH1 using *p*-nitrophenyl (*p*-NP) esters. The hydrolase activities are shown relative to the activity toward *p*-NB. (**D**) Regioselectivity of *La*SGNH1 was studied using 1-naphthyl acetate (1-NA), 1-naphthyl butyrate (1-NB), and 2-naphthyl acetate (2-NA). The hydrolase activities are shown relative to the activity toward 1-NA. (**E**,**F**) Detection of fluorescence due to the formation of 4-methylumbelliferone (4-MU) by *La*SGNH1. All experiments were performed at least in triplicate.

Recombinant *La*SGNH1 protein was purified by an immobilized Ni^2+^-affinity column to near homogeneity (Figure 3A). The molecular mass of *La*SGNH1 is similar to those of other SGNH family esterases, such as a thermostable and alkaline GDSL-motif esterase from *Bacillus* sp. K91 [16] or Lip2 from *Monascus purpureus* M7 [27]. However, it is smaller than the mass of a cold-adapted 36 kDa GDSL family esterase from *Photobacterium* sp. J15 [28]. In native polyacrylamide gel electrophoresis (PAGE), *La*SGNH1 showed a diffuse pattern (Figure 3B). The hydrolytic activity of *La*SGNH1 was analyzed using *p*-nitrophenyl esters of different chain lengths. As shown in Figure 3C, *La*SGNH1 showed high activities for short-chain substrates, such as *p*-nitrophenyl acetate (*p-*NA) and *p*-nitrophenyl butyrate (*p-*NB) (Figure 3C). However, very little activity was observed for *p*-nitrophenyl decanoate (*p-*ND) or *p*-nitrophenyl phosphate (*p*-NPP). This strong preference for short-chain *p*-nitrophenyl esters was also observed for other SGNH family members, such as an SGNH hydrolase from *Listeria innocua* 11262 [20] or SGNH hydrolases from *Sinorhizobium meliloti* [19,29]. When naphthyl esters were used as substrates, the highest activity was observed with 1-naphthyl acetate (1-NA) (Figure 3D). *La*SGNH1 showed regioselectivity, exhibiting only 25% activity toward 2-naphthyl acetate (2-NA) compared to 1-NA. Similar substrate specificity was observed in other members of the SGNH esterase family [23,24,28]. As shown in Figure 3E,F, strong fluorescence was observed for 4-methylumbelliferone (4-MU) acetate and *La*SGNH1, but not for 4-MU phosphate and *La*SGNH1.

**Figure 4:**
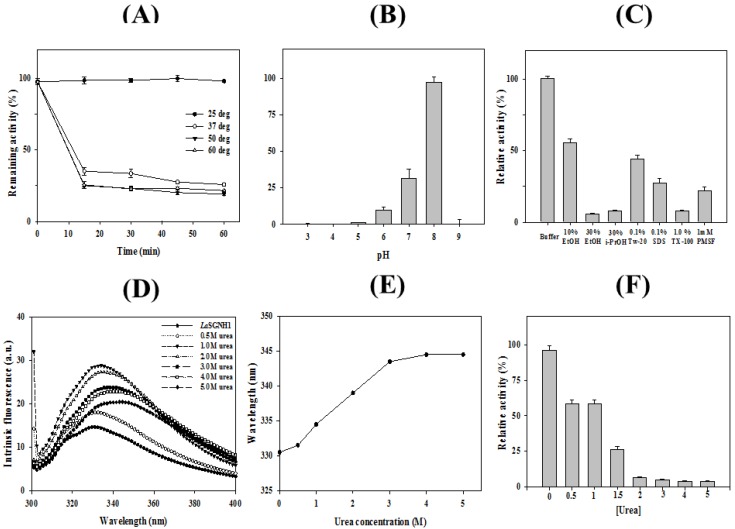
Stability of *La*SGNH1. (**A**) Thermal stability of *La*SGNH1. The residual activity of *La*SGNH1 was measured during incubation for 1-h. (**B**) The pH stability of *La*SGNH1 was studied at a pH from 3 to 10. (**C**) Chemical stability of *La*SGNH1 was studied against various chemicals. (**D**,**E**) Urea-induced unfolding of *La*SGNH1. Fluorescence was monitored after 1-h of incubation in from 1 to 5 M urea. A red-shift of λ_max_ from 330 to 344 nm was detected. (**F**) Activity of *La*SGNH1 in the different concentrations of urea. All experiments were performed at least in triplicate.

Thermostability of *La*SGNH1 was investigated over a temperature range from 25 to 60 °C (Figure 4A). Enzyme activity of *La*SGNH1 did not change significantly after 1-h of incubation at 25 °C. However, *La*SGNH1 showed only ~40% of initial activity after 15 min of incubation at 37 °C. Similarly, cinnamoyl esterases from Lactobacilli and Bifidobacteria showed an optimum temperature of 20–30 °C [30]. However, *La*SGNH1 showed lower thermostability compared to other SGNH family esterases, such as an SGNH-type esterase (*Lp*SGNH1) from *Lactobacillus plantarum* WCFS1 [12], a 7-aminocephalosporanic acid deacetylase [15], an alkaline SGNH hydrolase (Est19) from *Bacillus* sp. K91 [16], an SGNH hydrolase (LI22) from *Listeria innocua* [19], and an oligomeric SGNH-arylesterase from *Sinorhizobium meliloti* [20]. In addition, other esterases from *L. acidophilus* showed higher thermostability compared to *La*SGNH1. For example, an acetylesterase (*La*AcE) from *L. acidophilus* was shown to be stable at 40 °C for 1-h [31]. Moreover, no detectable activity loss of a feruloyl esterase from *L. acidophilus* was observed after a 2-h incubation at 37 °C [32].

In addition, *La*SGNH1 displayed its maximal activity at pH 8.0, whereas ~30% of this maximal activity was observed at pH 7.0 (Figure 4B). This optimum pH is similar to other SGNH family esterases, such as an esterase gene (Tlip) from *Thauera* sp. [14] or an SGNH hydrolase (LI22) from *Listeria innocua* [19]. Furthermore, other esterases from *L. acidophilus* showed the optimum pH of 7.0–8.0 such as a cinnamoyl esterase [30] or LaAcE [31].

As shown in Figure 4C, *La*SGNH1 retained ~65% of its initial activity in the presence of 10% ethanol and ~40% of its activity in the presence of 0.1% Tween 20. In contrast, the addition of 1.0% Triton X-100 resulted in less than 10% of its original activity. In the presence of 30% ethanol, *La*SGNH1 retained only 10% of its initial activity (Figure 4C). The chemical stability of *La*SGNH1 against urea was investigated by monitoring the intrinsic fluorescence changes. In the native state, *La*SGNH1 exhibited a λ_max_ at 330 nm, indicating that the tryptophan residues of *La*SGNH1 were located in the hydrophobic interior (Figure 4D,E). However, a red shift of λ_max_ to 344 nm was observed with a noteworthy increase of fluorescence intensity at 5 M urea. In contrast, the addition of 2.0 M urea resulted in almost complete loss of *La*SGNH1 activity (Figure 4F).

### 2.3. Homology Modeling and Substrate Analysis of LaSGNH1

**Figure 5:**
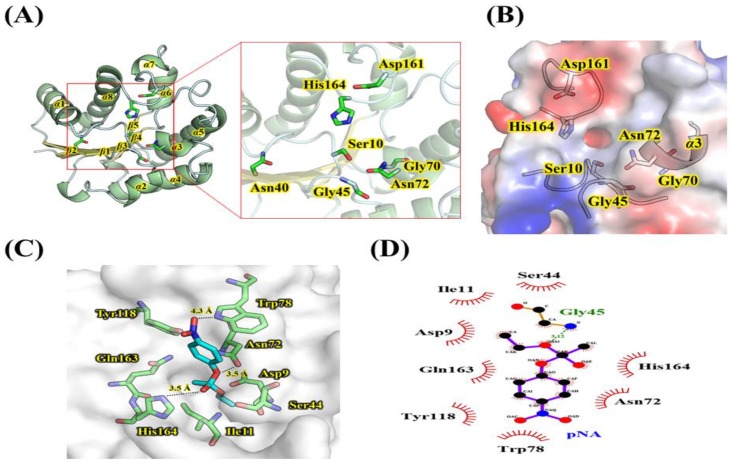
Homology modeling of *La*SGNH1. (**A**) Ribbon representation of *La*SGNH1. The substrate binding pocket is also shown in the square and important residues for catalysis are shown as sticks. (**B**) Electrostatic potential diagram of substrate-binding regions of *La*SGNH1. (**C**) Modeling of *p*-nitrophenyl acetate (*p*NA, cyan) in the substrate-binding pocket of *La*SGNH1. The amino acid residues interacting with *p*NA are shown as sticks (green). (**D**) LigPlot analysis of *p*-nitrophenyl acetate in the substrate-binding pocket of *La*SGNH1.

A structural model of *La*SGNH1 was constructed based on the crystal structure of lipase/acylhydrolase from *Enterococcus faecalis* (PDB I.D.: 1YZF). The putative catalytic triad of Ser^10^, Asp^161^, and His^164^ are positioned close to the outer solvent available surfaces (Figure 5A). Three amino acids, Gly^45^, Gly^70^, and Asn^72^, were identified to control the entrance of substrates toward the catalytic triad via noncovalent interactions (Figure 5B). These resides are also highly conserved in SGNH family esterases (see also Figure 1C). In molecular docking analysis, Asn^72^, Tyr^118^, and Gln^163^ were shown to stabilize the *p*-nitrophenol ring (Figure 5C,D). In addition, the backbone nitrogen of Gly^163^ is involved in the stabilization of an oxyanion hole.

**Figure 6:**
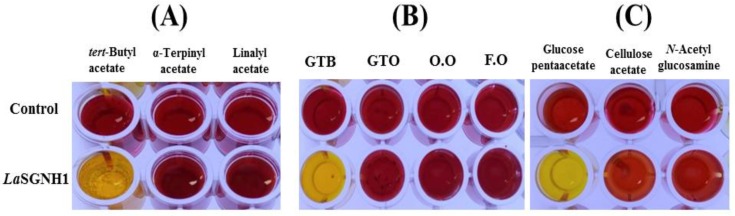
Hydrolysis of various substrates by *La*SGNH1. A pH shift assay was performed for (**A**) tertiary alcohol esters (TAEs), including *tert*-butyl acetate, α-terpinyl acetate, and linalyl acetate, (**B**) glyceryl esters, including glyceryl tributyrate (GTB) and glyceryl trioleate (GTO), and oils, including olive oil (O.O.) and fish oil (F.O.), and (**C**) acetylated carbohydrates, including glucose pentaacetate, cellulose acetate, and *N*-acetyl-glucosamine. The hydrolysis reaction changed the color of the solution from red to yellow.

The hydrolytic properties of *La*SGNH1 towards a wide range of substrates were studied using a colorimetric assay [33,34]. The ability of *La*SGNH1 to hydrolyze tertiary alcohol esters (TAEs) was investigated using *tert*-butyl acetate, α-terpinyl acetate, and linalyl acetate. As shown in Figure 6A, *La*SGNH1 was able to effectively hydrolyze *tert*-butyl acetate, but not α-terpinyl acetate nor linalyl acetate. Additionally, significant hydrolytic activity of *La*SGNH1 was only detected for glyceryl tributyrate, which was indicated by the yellow color of the solution (Figure 6B). In addition, strong hydrolytic activity of *La*SGNH1 for glucose pentaacetate was observed, although very little activity was observed in the presence of cellulose acetate or *N*-acetylglucosamine (Figure 6C). The preference of *La*SGNH1 for small-size substrates could be explained by the restricted dimensions of the substrate-binding pocket [35].

### 2.4. Immobilization of LaSGNH1

**Figure 7:**
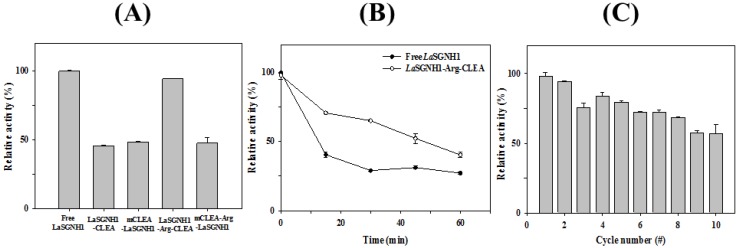
Immobilization of *La*SGNH1. (**A**) Immobilization efficiency of free *La*SGNH1, *La*SGNH1- crosslinked enzyme aggregates (CLEA), mCLEA-*La*SGNH1, *La*SGNH1-Arg-CLEA, and mCLEA-Arg-*La*SGNH1. (**B**) Thermal stability of free *La*SGNH1 and *La*SGNH1-Arg-CLEA. (**C**) Reusability of *La*SGNH1-Arg-CLEA. The reaction was repeated for 10 cycles after each washing step. All assays were performed at least in triplicate.

Enzyme immobilization, which could provide low cost, fast recovery, and high product yields, is widely used in industrial applications [36,37]. In previous reports, immobilized SGNH family esterases were shown to have better thermal stability, chemical stability, and recycling ability than free enzymes [12,18,19,29,35]. Specifically, cross-linked enzyme aggregates of LpSGNH1 displayed higher recycling ability and thermal stability than soluble LpSGNH1 [12]. In addition, enhanced thermal and chemical stability as well as good durability were observed in the crosslinked forms of LI22 [18] and Est24 [19]. Based on these studies, we immobilized *La*SGNH1 via chemical crosslinking. First, *La*SGNH1-crosslinked enzyme aggregates (CLEA) were prepared by precipitating *La*SGNH1 with ammonium sulfate and glutaraldehyde. In addition, arginine (Arg) was also included in the preparation of *La*SGNH1-Arg-CLEA, which was shown to be effective for the stability of immobilized enzymes [31,38]. Similarly, *La*SGNH1 was co-precipitated with magnetite Fe_3_O_4_ nanoparticles, and crosslinked using glutaraldehyde to obtain magnetic *La*SGNH1-CLEA (mCLEA-*La*SGNH1). Enzyme immobilization using magnetite Fe_3_O_4_ nanoparticles could be used for fast separation [39]. Among these four different immobilization approaches (*La*SGNH1-CLEA, *La*SGNH1-Arg-CLEA, mCLEA-*La*SGNH1, and mCLEA-Arg-*La*SGNH1), *La*SGNH1-Arg-CLEA showed the highest immobilization efficiency, which was comparable to that of free *La*SGNH1 (Figure 7A).

Next, the thermal stability of *La*SGNH1-Arg-CLEA was investigated for 1-h of incubation at 37 °C. As shown in Figure 7B, immobilized *La*SGNH1-Arg-CLEA retained ~70% of its original activity after 30 min, while the free *La*SGNH1 showed only 31% of its activity. Furthermore, the reusability of *La*SGNH1-Arg-CLEA was studied over 10 cycles. After each cycle, the *La*SGNH1-Arg-CLEAs were separated by centrifugation and washed for the next cycle. As shown in Figure 7C, *La*SGNH1-Arg-CLEA showed good recycling ability, retaining about 60% of the original activity even after the 10th cycle. Therefore, *La*SGNH1-Arg-CLEA showed good immobilization efficiency, enhanced thermal stability, and high reusability, which could be exploited to facilitate the applications of *La*SGNH1.

## 3. Materials and Methods

### 3.1. Reagents

DNA-modifying enzymes were obtained from New England BioLabs (Ipswich, MA, USA). DNA purification kits were obtained from Qiagen Korea (Daejon, Korea), and protein purification columns were purchased from GE Healthcare (Seoul, Korea). All other reagents were of analytical grade and were purchased from Sigma-Aldrich Korea (Yongin, Korea).

### 3.2. Bioinformatic Analysis

The primary sequences of *La*SGNH1 and related proteins were retrieved from the NCBI database. Multiple sequence alignments and sequence comparisons were carried out using Clustal Omega [40] and ESPript [41]. A phylogenetic tree was constructed by MEGA v. 7.0 using the neighbor-joining method with 2000 iterations [42]. A structural model of *La*SGNH1 was constructed based on the crystal structure of lipase/acylhydrolase from *Enterococcus faecalis* (PDB I.D.: 1YZF) using the SWISS-MODEL server. Molecular docking analysis was performed using flexible side chain methods and AutoDock Vina [43].

### 3.3. Cloning and Purification

*L. acidophilus* NCFM (KCTC 3145; Korean Collection for Type Cultures) were cultured in MRS medium (BD Difco, NJ, USA) and chromosomal DNA was purified using a DNeasy Tissue and Blood Kit (Qiagen, USA). The open reading frame of the *La*SGNH1 gene was amplified by polymerase chain reaction (PCR), and the PCR product was cloned into pQE-30 plasmid using *BamHI* and *XhoI*. After DNA sequencing, the recombinant plasmid (pET-*La*SGNH1) was transformed into *Escherichia coli* cells for protein expression of *La*SGNH1. *E. coli* cells were grown until the optical density (OD_600nm_) reached 0.6–0.8. After 1 mM isopropyl-β-D-1-thiogalactoside induction for 4 h at 37 °C, cells were centrifuged and resuspended in lysis buffer (20 mM Tris-HCl pH 8.0, 50 mM NaCl, 1 mM EDTA). After keeping on ice for 15 min, the cellular membrane was disrupted using a microtip (1-s pulse, 3-s pause, and 51% amplitude) in a Q500 sonicator (Terra Universal, Fullerton, CA, USA). After sonication, the supernatants were loaded onto a HisTrap HP column using an AKTA Prime Plus (GE healthcare, Chicago, IL, USA). The recombinant *La*SGNH1 protein was eluted with an imidazole gradient from 50 to 300 mM. After a washing process, the pooled fractions were desalted with a lysis buffer. Protein concentration was determined using a Biorad Protein assay kit (Bio-rad Laboratories, Chicago, IL, USA) and purified *La*SGNH1 was stored at −20 °C.

### 3.4. Biochemical Characterization of LaSGNH1

Substrate specificities of *La*SGNH1 were investigated using *p*-nitrophenyl (*p*-NP) esters and naphthyl esters. The amounts of released *p*-nitrophenol were measured at 405 nm using *p*-nitrophenyl acetate (*p-*NA), *p*-nitrophenyl butyrate (*p-*NB), *p*-nitrophenyl hexanoate (*p-*NH), *p*-nitrophenyl octanoate (*p-*NO), *p*-nitrophenyl decanoate (*p-*ND), and *p*-nitrophenyl phosphate (*p-*NPP) [44,45]. For naphthyl esters, the formation of naphthol was monitored at 310 nm. The standard assay solution included 50 µM substrate in 20 mM Tris-HCl (pH 8.0) with 1 µg of *La*SGNH1, and the assay ran for 10 min at 25 °C. All spectroscopic analyses were carried out using an Epoch 2 Microplate Spectrophotometer (BioTek, Winooski, VT, USA). Hydrolysis of 4-MU acetate or phosphate was also measured using a Jasco FP-8200 spectrofluorometer (Jasco, Japan) or an Eppendorf tube containing *La*SGNH1 in a UV illumination box.

The thermostability and pH stability of *La*SGNH1 were investigated at different temperatures ranging from 25 to 60 °C and across a pH range of 3.0 to 10.0. Effects of chemicals (10% ethanol, 30% ethanol, 30% *iso*-propanol, 0.1% Tween 20, 0.1% SDS, 1.0% Triton X-100, 1 Mm PMSF, and urea (from 0 to 5 M)) on the activity of *La*SGNH1 were investigated after 1-h incubation using *p*-nitrophenyl butyrate (*p*-NB) as a substrate, and the enzyme activity of *La*SGNH1 in buffer alone was defined as 100%. For intrinsic fluorescence spectra, the emission spectra from 300 to 400 nm were measured after excitation at 295 nm. All spectra were measured with a scan speed of 500 nm·min^−1^ and a 2 nm bandwidth using a Jasco FP-8200 spectrofluorometer.

For pH-indicator-based colorimetric assays, 1 µg of *La*SGNH1 was added to a phenol-red-containing substrate solution. The substrates included lipids (glyceryl tributyrate, glyceryl trioleate, olive oil, and fish oil), tertiary alcohol esters (tertiary butyl acetate, α-terpinyl acetate, and linalyl acetate), and acetylated carbohydrates (glucose pentaacetate, cellulose acetate, and *N*-acetyl-glucosamine) [33,45].

### 3.5. Immobilization of LaSGNH1

For the preparation of cross-linked enzyme aggregates (CLEA), 0.5 mg·mL^−1^ of *La*SGNH1 was co-precipitated with 70% ammonium sulfate with glutaraldehyde, incubated overnight, and centrifuged. The pellet (*La*SGNH1-CLEA) was resuspended and washed extensively until no significant enzyme activity was detected in the supernatant. Addition of Arg and Fe_3_O_4_ magnetic nanoparticles for the preparation of *La*SGNH1-Arg-CLEA, mCLEA-*La*SGNH1, and mCLEA-Arg-*La*SGNH1 was carried out as described previously [31,45]. For thermal stability, *La*SGNH1-Arg-CLEA and free *La*SGNH1 were incubated at 37 °C for 1-h. For the reusability experiments, *La*SGNH1-Arg-CLEA was reused after extensive washing in subsequent cycles.

## 4. Conclusions

Although SGNH family esterases have attracted interest due to their potential applications, there remains little information about this family from lactic acid bacteria. Here, a novel SGNH family esterase (*La*SGNH1) from *L. acidophilus* NCFM was identified, characterized, and immobilized. The novel properties of *La*SGNH1 could make it a promising candidate for the food, cosmetics, pharmaceutical, and biofuel industries. In addition, this study could help us to better understand the SGNH family esterases, although the physiological role of *La*SGNH1 has not yet been revealed. Further studies on *La*SGNH1, including mutagenesis of key residues, structural determination, and formation of the enzyme–substrate complex, will be necessary to further our understanding of this *La*SGNH1 enzyme.
